# Removal of a Piece of Gun Barrel from the Outer Surface of the Third Rib

**Published:** 1857-05

**Authors:** A. W. Griggs

**Affiliations:** Newnan, Ga.


### ARTICLE III.

*Removal of a piece of Gun Barrel from the outer surface of the Third Rib.* By A. W. Griggs, M. D., Newnan, Ga., April 10th, 1857.

Professor Logan,

*Dear Sir*,—On the morning of the 16th of September last, I called in the office of Dr. L. W. Pierce, about six miles from Newnan, on the road leading to Moore's Ferry. Soon afterwards Mr. J. G. Giles, of Carroll, the adjoining county, entered the room, having walked several miles from home beyond the river to consult my friend in reference to an injury which he had received, on the night of the 5th of December, 1855, by the explosion of a shot gun, the contents of which he was attempting to discharge at an owl that was among the poultry. At the fire of the gun the barrel split in half, longitudinally from the breach, to the extent of about five inches, and the left half was blown away, inflicting a severe wound on the left side of the chest, a few inches in front of the axilla. He informed us that a couple of medical gentlemen were summoned, and after examination, the edges of the wound were drawn together by adhesive straps, and he was left. We were informed that the union by the first intention took place without any difficulty, but that a dull aching pain between the axilla and the sternum gave the patient but little rest. That in the course of a few weeks, a hard and extremely painful ridge in that direction formed, which finally pointed and opened an inch to the sternal side of the original cut, and a great quantity of thick purulent matter was discharged. That lately; a second opening had appeared, half an inch to the right of the former. The patient began to suspect that something was concealed in the wound. Upon slight examination, we thought that there was either caries of the rib, or a piece of gun barrel lodged beneath the pectoral muscles, or probably both. I introduced a probe into the fistulous opening next to the axilla, and moved the point gently forward and downward, it infringed directly against some smooth slick metallic substance. The probe being withdrawn, it was then passed into the same opening and out at the other. Mr. G. consented that we might make a short incision down upon the probe, which was accordingly done, but being insufficient for our purpose, the incision was continued toward the axilla to the distance of an inch. Whereupon we discovered a large piece of iron, which he was anxious to have removed.

We had no instruments save a dissecting case. Drs. Smith & Bruce were a short way off, and were invited to attend and assist us; neither had his pocket case. They coincided in our opinion, that there would be but little or no risk in dividing the muscles under which the iron had been plunged. Chloroform was administered, and with a small bistoury, I proceeded to make an incision from the second opening to the sternum, using little traction; a piece of the barrel $4\frac{7}{8}$ inches long, and weighing $2\frac{1}{2}$ oz., was removed from the denuded rib. The concavity which was part of the original bore of the barrel, precisely fitted over the rib, and the piece was so curved by the lateral pressure of the powder during its expansion from combustion, as to be brought in close adaptation with the convexity of the rib; the lateral edges dipping into the intercostal spaces above and below, formed by the contiguous ribs with the third. Slight hemorrhage occurred from the division of some of the ramifications of the external mammary artery; ligatures were applied, and hemorrhage ceased. No perceptible anatomical changes had taken place in the parts, except about the openings, which were soft, pulpy, purple, and nearly insensible. The edges of the wound being brought together, sufficient opening was left for the evacuation of the unhealthy accumulations from the diseased structures. Of course, union by the first intention, only took place in the part unaltered by disease—the other healing by granulation. The patient, at the time of the operation, was very much emaciated—of a sallow complexion. He had made a crop with his own hands the preceding season. He visited my office a few days ago. He is now looking well, and has gained fifty pounds in weight since the operation.

